# Factors Associated With Job Satisfaction of Frontline Medical Staff Fighting Against COVID-19: A Cross-Sectional Study in China

**DOI:** 10.3389/fpubh.2020.00426

**Published:** 2020-08-04

**Authors:** Xiaoyan Yu, Yuxin Zhao, Yuxi Li, Chao Hu, Huilan Xu, Xianmei Zhao, Jin Huang

**Affiliations:** ^1^Department of Social Medicine and Health Management, Xiangya School of Public Health, Central South University, Changsha, China; ^2^Department of Clinical Nursing, The Second Xiangya Hospital of Central South University, Changsha, China; ^3^Department of Health Toxicology, Xiangya School of Public Health, Central South University, Changsha, China; ^4^High School of National University of Defense Technology, Changsha, China

**Keywords:** COVID-19, frontline medical staff, job satisfaction, influencing factors, workload

## Abstract

**Purpose:** The current research on frontline medical staff in China fighting against COVID-19 has not yet addressed job satisfaction. The purpose of this study is to investigate the job satisfaction of those who were sent to support Hubei province, China, or worked in local designated hospitals, and then analyze the associated influencing factors.

**Materials and Methods:** A total of 455 medical staff who worked at the frontline of the prevention and control of COVID-19 in Hubei province was selected using simple random sampling. They were asked to fill out a self-developed general information questionnaire as well as the Minnesota Satisfaction Questionnaire (MSQ), from 10 January to 10 March 2020.

**Results:** The average job satisfaction score of the participants was 82.58 ± 11.11. The influencing factors include education (*P* = 0.002), years of work experience *(P* = 0.006), anti-epidemic work duration (*P* = 0.048), daily sleep duration (*P* < 0.001), and the form of participation (*P* < 0.001).

**Conclusions:** This study, for the first time, measures the job satisfaction of frontline medical staff in fighting against COVID-19 in China. The job satisfaction of frontline medical staff was at a “relatively decent” level, higher than the previous similar measures among medical staff. Related management departments should further improve the job satisfaction of frontline medical staff by meeting their reasonable demands, strengthening the emergency response and practical operation training of junior staff, and ensuring their ample time for sleep and rest. This study is of great reference value for improving the job satisfaction level of frontline medical staff during public health emergencies, developing medical staff security policies, and promoting the establishment of emergency response teams.

## Introduction

The novel coronavirus pneumonia (COVID-19) is a new infectious disease that is highly contagious (1). Since its initial discovery in Wuhan, Hubei province, in December 2019, the disease has broken out rapidly both across China and around the world. On January 30, 2020, COVID-19 was listed as a public health emergency of international concern by the World Health Organization (WHO) (2). Up to April 5, 2020, 208 countries and regions have reported a total of 1,093,349 confirmed cases of COVID-19 and 58,620 deaths (3), whereas the figures in China are, respectively, 81,669 and 3,329 (4).

During the early stage of the fight against COVID-19, all provinces in China promptly responded to the widespread transmission of the disease, deciding lists of designated hospitals and fever clinics for the treatment of COVID-19. In addition, numerous medical staff joined the frontline of the prevention and control of COVID-19 and participated in the diagnosis, screening, inspection, examination, transfer, treatment, nursing, epidemiological investigation, medical observation, specimen collection, pathogen detection, pathological examination, and pathological autopsy of confirmed and suspected cases (5). Between January and March 2020, more than 40,000 medical personnel were sent to Hubei province to aid in the treatment of COVID-19. These medical teams, which often consisted of staff from different medical institutions, participated in epidemic prevention and control by taking control of entire wards and mostly worked at designated hospitals and mobile cabin hospitals in Wuhan city and Huanggang city. As the core of the fight against COVID-19, these supportive medical teams were assigned the mission of saving patients' lives as well as preventing and controlling the pandemic outbreak. However, owing to the sudden outbreak of the COVID-19, the number of infections considerably increased in a short period of time. Therefore, frontline medical staff faced more intense workloads (6–8), higher risks of infection (9), and heavier physical (10–12) and mental stresses (13–16), which challenged their job satisfaction (17). The current research on frontline medical staff in this fight in China mainly includes skin conditions (18), sleep disturbances (19, 20), mood symptoms (21), anxiety (22), depression (23), exercise rehabilitation (24), and other aspects. However, the job satisfaction of frontline medical staff in China was not involved.

Job satisfaction measures an employee's cognition and evaluation, mental status, and emotional experience of their job as well as all related aspects or facets (25). During the epidemic, some scholars studied the job satisfaction of manufacturing workers (26), and some have also explored the job satisfaction of healthcare staff in Iran (27). The job satisfaction of frontline medical staff is directly associated with the implementation and effectiveness of strategies for the prevention and control of major crises. Therefore, this study is of great research significance. To ensure that frontline medical staff can be fully devoted to the epidemic prevention and control, the National Health Commission of the People's Republic of China issued the “Notice on Providing Full Security for Frontline Medical Staff and Their Families” on February 7, 2020, which required that the living, psychology, security, and their other needs should be guaranteed (28). After the fight for 2 months, the epidemic situation in China has gradually improved (4). So WHO announced that China had moved from the “containment phase” to the “mitigation phase.” However, the job satisfaction status of Chinese frontline medical staff during the fight against COVID-19 remains unexplored. Thus, there is a strong need to investigate every aspect that influences the performances of the medical staff.

This study focuses on a category of workers at high risk for COVID-19, and for the first time, the job satisfaction of China's frontline medical staff has been surveyed. The surveyed content included job characteristics related to the fight against COVID-19 as well as some demographic variables that were statistically significant in previous medical staff job satisfaction research to analyze the associated influencing factors. This study is of great reference value for improving the job satisfaction level of frontline medical staff during public health emergencies, developing medical staff security policies, and promoting the establishment of emergency response teams.

## Materials and Methods

### Study Design and Data Collection

Between March 25 and April 5, 2020, members of three medical teams that were sent to Hubei to aid the prevention and control of COVID-19, as well as medical staff of a designated hospital for COVID-19 treatment, were selected through simple random sampling. A total of 455 frontline medical staff was then enrolled in the study. The inclusion criteria were as follows: (1) Medical staff who directly participated in the fight against COVID-19 by “contacting confirmed/suspected COVID-19 cases or their specimens,” and (2) Those who voluntarily participated in the study and provided their informed consent. The exclusion criteria were as follows: (1) Non-frontline medical staff, and (2) Those who could not complete the questionnaire.

The studies were reviewed and approved by the Ethics Committee of Xiangya School of Public Health of Central South University. The researchers introduced the purpose and significance of the study to the participants through interviews, phone calls, or WeChat, and then sent them an electronic questionnaire and provided them with unified instructions on the completion of the measure. A total of 468 questionnaires were sent out, and 468 responses were collected. Both the response and completion rates of the questionnaire were 100%. Thirteen questionnaires completed by non-frontline medical staff were excluded. Thus, a total of 455 valid questionnaires were collected, representing an effective response rate of 97%.

### Demographic Variables and Working Characteristics

By reviewing previous literature and consulting hospital management specialists as well as some frontline anti-epidemic medical staff, our research team developed a general information questionnaire covering aspects that could affect the job satisfaction of frontline medical staff in the prevention and control of COVID-19. The questionnaire contained items of demographic characteristics, including gender, age, education, and marital status; and job characteristics, including occupation, years of work experience, job title, working unit, the form of participation, anti-epidemic work duration, daily sleep duration during the anti-epidemic work, and location of the anti-epidemic work.

### Measurement of Job Satisfaction About Frontline Medical Staff

Job satisfaction was surveyed by the Minnesota Satisfaction Questionnaire (MSQ), which was initially developed by Weiss in 1967 (29). In this study, its Chinese version (30) was adopted to measure the job satisfaction of frontline anti-epidemic medical staff. MSQ is commonly used to measure the job satisfaction of employees in China. It contains a total of 20 items, each of which is scored on a Likert scale of 1–5, with 1 denoting “Not satisfied” and 5, “Extremely satisfied.” The total score of the MSQ ranges from 20 to 100, with a higher total score indicating a higher global job satisfaction. The score of each item is further divided into three levels, with 1.0–3.0 indicating low satisfaction, 3.1–4.0 indicating moderate satisfaction, and 4.1–5.0 indicating high satisfaction. The job satisfaction of the respondent is then categorized as follows: not satisfied (20–30); somewhat satisfied (31–50); satisfied (51–70); very satisfied (71–90); and extremely satisfied (91–100). The Cronbach's α of the questionnaire was 0.962 in this study.

### Statistical Methods

IBM SPSS Statistics 23.0 was used to establish databases and perform statistical analyses. The demographic characteristics, job characteristics, and job satisfaction of frontline medical staff in the prevention and control of COVID-19 were statistically described. In the descriptive analysis, measurement data were expressed as *x̄* ± *s*, whereas count data were expressed as frequency or percentage. Univariate analysis and *post-hoc* tests were performed on comparison of the job satisfaction of frontline medical staff with different demographic and job characteristics (pairwise comparison was conducted using the *t*-test, and comparison between multiple groups was conducted using the analysis of variance). Multivariate linear regression was utilized to analyze the influencing factors of job satisfaction. The significance level was set at α = 0.05.

## Results

### Demographic and Job Characteristics

As is seen in Table 1, of the 455 frontline anti-epidemic medical staff, most were women (70.5%) and aged 30–40 years (55.4%). Most participants were undergraduate (66.6%), followed by postgraduate or higher (26.2%). In addition, the majority of the participants were married (67.7%). The frontline medical staff surveyed in this study was predominantly nurses (70.3%), most of whom had either a junior (41.3%) or an intermediate title (45.3%). 97.6% of individuals were the self-participation medical staff. In addition, 62.4% of the participants worked in the intensive care unit, and 63.7% had continuously worked for over a month.

**Table 1:** Demographic and job characteristics of frontline medical staff fighting against COVID-19.

**Item**	**Category**	***N***	**(%)**
Gender	Male	134	29.5
	Female	321	70.5
Age (years)	<30	156	34.3
	30–40	252	55.4
	>40	47	10.3
Education	≤ College	33	7.2
	Undergraduate	303	66.6
	≥Postgraduate	119	26.2
Marital status	Single	141	31
	Married	308	67.7
	Others	6	1.3
Occupation	Physician	115	25.3
	Nurse	320	70.3
	Screening technician	20	4.4
Work experience	≤ 6 years	150	33
	6–12 years	149	32.7
	>12 years	156	34.3
Technical title	Junior	188	41.3
	Intermediate	206	45.3
	Senior	61	13.4
Working unit	Screening ward	22	4.8
	General ward	149	32.8
	Intensive care unit	284	62.4
Form of participation	Self-participation	444	97.6
	Hospital arrangements	11	2.4
Working duration	<2 weeks	5	1.1
	2 weeks to 1 month	160	35.2
	>1 month	290	63.7
Daily sleep duration	≤ 6 h	188	41.3
	≥ 7 h	267	58.7
Location	Hubei province	188	41.3
	Outside Hubei province	267	58.7

### Individual Job Satisfaction Scores

The individual job satisfaction scores are shown in Table 2. The five items with the highest average scores were, respectively, relationships with colleagues (4.38 ± 0.596), work fulfillment (4.33 ± 0.660), contribution to others (4.33 ± 0.605), work recognition (4.28 ± 0.656), and moral value (4.26 ± 0.709). Meanwhile, of the 20 items, the one with the lowest job satisfaction score was the opportunity for promotion (3.71 ± 0.873). All items had a score of 3 and above, indicating that the satisfaction level of the participants was above average in all aspects.

**Table 2:** Job satisfaction of frontline medical staff.

**Item**	**Job satisfaction level** ***n*** **(%)**					**Job satisfaction score (‘x ± s, point)**
	**Not**	**Somewhat**	**Satisfied**	**Very**	**Extremely**	
	**satisfied**	**satisfied**		**satisfied**	**satisfied**	
**Intrinsic satisfaction**						
Work intensity	6 (1.3)	29 (6.4)	62 (13.6)	275 (60.4)	83 (18.3)	3.88 ± 0.824
Work independence	6 (1.3)	11 (2.4)	24 (5.3)	292 (64.2)	122 (26.8)	4.13 ± 0.722
Diversity of work content	6 (1.3)	16 (3.5)	58 (12.8)	264 (58.0)	111 (24.4)	4.01 ± 0.795
Social status of work role	4 (0.9)	8 (1.7)	24 (5.3)	298 (65.5)	121 (26.6)	4.15 ± 0.668
Moral value	5 (1.1)	6 (1.3)	22 (4.8)	253 (55.6)	169 (37.2)	4.26 ± 0.709
Work stability	5 (1.1)	5 (1.1)	27 (5.9)	259 (56.9)	159 (35.0)	4.24 ± 0.705
Contribution to others	2 (0.4)	3 (0.7)	12 (2.6)	264 (58.0)	174 (38.3)	4.33 ± 0.606
Authority of instructing others	3 (0.7)	6 (1.3)	38 (8.4)	280 (61.5)	128 (28.1)	4.15 ± 0.674
Demonstration of individual ability	5 (1.1)	9 (2.0)	33 (7.3)	264 (58.0)	144 (31.6)	4.17 ± 0.734
Freedom to make independent decisions	3 (0.7)	15 (3.3)	46 (10.1)	290 (63.7)	101 (22.2)	4.04 ± 0.717
Work creativity	6 (1.3)	12 (2.6)	50 (11.0)	276 (60.7)	111 (24.4)	4.04 ± 0.760
Work fulfillment	2 (0.4)	5 (1.1)	22 (4.9)	239 (52.5)	187 (41.1)	4.33 ± 0.661
**Extrinsic satisfaction**						
Subordinate relationship	6 (1.3)	12 (2.7)	27 (5.9)	267 (58.7)	143 (31.4)	4.16 ± 0.757
Leadership management skills	5 (1.1)	11 (2.4)	31 (6.8)	268 (58.9)	140 (30.8)	4.16 ± 0.740
Workplace policies and systems	4 (0.9)	7 (1.5)	35 (7.7)	258 (56.7)	151 (33.2)	4.20 ± 0.715
Salaries and allowances	4 (0.9)	24 (5.3)	80 (17.6)	243 (53.4)	104 (22.8)	3.92 ± 0.831
Promotion opportunities	6 (1.3)	24 (5.3)	148 (32.5)	192 (42.2)	85 (18.7)	3.72 ± 0.874
Working environment and conditions	4 (0.9)	26 (5.7)	34 (7.5)	273 (60.0)	118 (25.9)	4.04 ± 0.801
Relationship with colleagues	2 (0.4)	2 (0.4)	9 (2.0)	248 (54.5)	194 (42.7)	4.38 ± 0.597
Work recognition	2 (0.4)	6 (1.3)	22 (4.9)	258 (56.7)	167 (36.7)	4.28 ± 0.656

### Univariate Analysis of Job Satisfaction

We compared the job satisfaction scores among frontline medical staff with various demographic and job characteristics and found statistically significant differences (*P* < 0.05) among different groups for the following: education, work experience, the form of participation, anti-epidemic work duration, and daily sleep duration. Details of the results are shown in Table 3. After a further comparison between every two groups by LSD showed that substantially higher job satisfaction compared with those with a postgraduate and higher (*P* = 0.037). In terms of work experience, staff who had worked for over 12 years demonstrated a higher satisfaction than those with <6 years (*P* = 0.042). In terms of the form of participation, self-participation exhibited a significantly higher satisfaction than those arranged by the hospital (*P* < 0.001). In terms of daily sleep duration, staff with over 7 h sleep daily exhibited a considerably higher satisfaction than those with <6 h (*P* < 0.001). Lastly, in terms of anti-epidemic work duration, those who had worked between 2 weeks and 1 month displayed a higher job satisfaction compared with the other two groups (*P* = 0.021).

**Table 3:** Comparison of job satisfaction scores among frontline medical staff with various demographic and job characteristics (*x̄* +*s*).

**Item**	**Category**	**MSQ score**	***t/F***	***P***
Gender	Male	81.69 ± 11.40	−1.105	0.27
	Female	82.96 ± 10.98		
Age (years)	<30	82.08 ± 11.04	0.252	0.777
	30–40	82.87 ± 11.68		
	>40	82.70 ± 7.87		
Education	≤ College	84.48 ± 9.83	3.329	0.037
	Undergraduate	83.23 ± 11.04		
	≥Postgraduate	80.40 ± 11.39		
Marital status	Single	81.05 ± 10.78	2.317	0.1
	Married	83.35 ± 11.18		
	Others	79.50 ± 11.11		
Occupation	Physician	81.25 ± 10.94	1.384	0.252
	Nurse	83.15 ± 11.27		
	Screening technician	81.25 ± 8.97		
Work experience	≤ 6 years	80.91 ± 11.96	3.182	0.042
	6–12 years	82.70 ± 11.48		
	>12 years	84.09 ± 9.64		
Technical title	Junior	82.65 ± 11.66	0.014	0.986
	Intermediate	82.59 ± 11.31		
	Senior	82.38 ± 8.55		
Working unit	Screening ward	79.68 ± 8.17	2.173	0.115
	General ward	83.95 ± 8.84		
	Intensive care unit	82.09 ± 12.26		
Form of participation	Self-participation	83.04 ± 10.15	5.695	<0.001
	Hospital arrangements	64.36 ± 25.80		
Working duration	<2 weeks	81.60 ± 7.02	3.887	0.021
	2 weeks to 1 month	84.54 ± 9.63		
	>1 month	81.52 ± 11.78		
Daily sleep duration	≤ 6 h	80.12 ± 13.11	−4.034	<0.001
	≥ 7 h	84.32 ± 9.08		
Location	Hubei province	82.93 ± 10.18	0.549	0.583
	Outside Hubei province	82.34 ± 11.73		

### Multiple Linear Regression Analysis of Job Satisfaction

A multiple linear regression model was established by taking the total job satisfaction scores of the participants as the dependent variable, the five statistically significant variables determined in the univariate analysis as the independent variables, and other factors (e.g., gender, age, marital status, occupation, job title, anti-epidemic working environment, and the province of the anti-epidemic work) as the covariate variables. Subsequently, the influencing factors of the job satisfaction of frontline anti-epidemic medical staff were determined. Values assigned to the five independent variables are listed in Table 4. All these variables were included in the regression equation. The results suggested that education, anti-epidemic work duration, and the form of participation was served as negative predictors for the job satisfaction of frontline medical staff, whereas work experience and daily sleep duration served as positive predictors. In addition, as is shown in Table 5, education, years of work experience, anti-epidemic work duration, average daily sleep duration, and the form of participation could jointly explain 13.3% of all variations in the job satisfaction of them. Standardized partial regression coefficients indicated that the form of participation exhibited the largest influence on the job satisfaction of frontline medical staff.

**Table 4:** Variable assignment.

**Variable**	**Assignment**
Education	≤ College = 1, Undergraduate = 2, ≥Postgraduate = 3
Work experience	≤ 6 years = 1, 6–12 years = 2, >12 years = 3
Working time	<2 weeks = 1, 2 weeks to 1 month = 2, >1 month = 3
Average sleeping time	≤ 6 h = 1, ≥ 7 h = 2
Form of participation	Self-participation = 1, Hospital arrangements = 2

**Table 5:** Multivariate analysis of the job satisfaction of frontline medical staff (*n* = 455).

**Independent variable**	**Standardized partial**	***t***	***P***
	**regression coefficient**		
Education	−0.143	−3.175	0.002
Work experience	0.124	2.758	0.006
Anti-epidemic work duration	−0.088	−1.986	0.048
daily sleep duration	0.174	3.911	<0.001
Form of participation	−0.233	−5.269	<0.001

## Discussion

### The Present Situation of Job Satisfaction

Unprecedentedly, this study investigated the job satisfaction of frontline medical staff fighting against COVID-19. Our survey results suggested that the average job satisfaction score of 455 frontline medical staff who participated in the survey was 82.58 ± 11.11, indicating a “relatively decent” (30) level of satisfaction, higher than that reported in previous surveys among medical staff (31–34). In previous studies, the job satisfaction of Chinese medical staff normally used the Minnesota Satisfaction Questionnaire (MSQ). Ge et al. conducted a cross-sectional survey of 2100 Chinese community health workers from the two cities and found that the overall job satisfaction scores of these workers were 67.17 and 69.95, respectively (35). Li et al. carried out a large cross-sectional study on 2,873 anesthesiologists from 211 hospitals in Beijing, Tianjin, and Hebei, China, and found that the total score of job satisfaction of Chinese anesthesiologists was 65.3 ± 11.5 (36). Xiao et al. conducted a questionnaire to survey all physicians in emergency departments of three large general hospitals in China and found that the overall job satisfaction score of emergency physicians was 68.72 ± 10.90 (37). Zhou et al. cross-sectional survey of 32 tertiary psychiatric hospitals in 29 provincial capital cities in China found that the general job satisfaction score of 9,907 psychiatric nurses in China was 73.7 (38). It is clear that the job satisfaction score of frontline medical staff fighting against COVID-19 is higher than those staff mentioned above.

Relationships with colleagues, anti-epidemic work fulfillment, and work recognition were the items scoring the highest marks. This is probably because the hard work of frontline medical staff was widely recognized by society, and their needs were guaranteed by security policies during this public health emergency. To ensure that frontline medical staff can fully devote themselves to the prevention and control of COVID-19, the Chinese government issued a series of measures to improve their working conditions (5, 28, 39–41), including the following: allowances not subject to identities and job titles; provision of security in terms of the working environment, protective materials, and living demands; enhancement of protection and treatment; implementation of rotations and shifts; provision of psychological counseling; and introduction of reward policies by providing promotion opportunities as well as a commendation of outstanding personnel and groups. The introduction and implementation of these measures greatly elevated the professional value and social status of frontline medical staff and promoted their working enthusiasm, thereby improving their job satisfaction during the fight against COVID-19. In addition, as all associated treatment costs of COVID-19 were shouldered by the government (36), the financial burden on patients and their families was substantially alleviated, which facilitated a cooperative and trustful relationship between doctors and patients in designated hospitals. The trust and gratitude from the patients, family members, and society, to some extent, improved the professional fulfillment and job satisfaction of frontline medical staff.

Items that had a low job satisfaction score included work intensity and salaries. This mainly lies in two reasons. On the one hand, the number of patients rapidly increased during the early stage of the outbreak. Therefore, there were significant shortages of wards, treatment beds, and medical specialists, which led to considerable increases in the workload and work intensity of frontline medical staff. On the other hand, owing to close contact with COVID-19 patients, they faced the risk of infection at all times, which endangered their mental and physical health.

### Analysis of Influencing Factors

The univariate analysis results suggested that, unlike in previous surveys, the job satisfaction of medical staff at the frontline of the prevention and control of COVID-19 did not show statistically significant differences between participants with different job characteristics, such as their original locations, occupations, and working environment. This outcome is possibly related to the fact that due to the temporary team establishment, team members aim to defeat the epidemic regardless of their technical title and leader-member relation. They form a “comrades-in-arms” relationship during the fight. Multiple linear regression analysis indicated that the factors influencing the job satisfaction of frontline anti-epidemic medical staff includes education, years of work experience, anti-epidemic work duration, daily sleep duration, and the form of participation.

In terms of education, the higher the education levels of frontline medical staff, the lower their job satisfaction. This is probably because staff with a higher education level often had more extensive theoretical knowledge and, consequently, higher expectations for their professional knowledge (42). However, the complexity and difficulty of the actual prevention and control of COVID-19 resulted in a special working environment that reduced their sense of satisfaction. Another possible reason is that during the fight against COVID-19, staff were often assigned to identical positions and tasks that could not reflect the advantages of having higher educational degrees or realize self-fulfillment, thereby causing psychological falls to those with higher education (43, 44).

Meanwhile, years of work experience served as a positive predictor for the job satisfaction of staff surveyed in this study: the less the work experience is, the lower the job satisfaction is. This is possibly due to the lack of professional skills. For a vast majority of staff, this was their first time facing a major public health emergency, which posed several challenges to their professional skills, occupational protection, and psychological tolerance. On the contrary, medical staff with longer work experience could make judgments more rapidly owing to their rich clinical experience, thereby making their jobs easier (45).

Our survey also found that medical staff who had worked between 2 weeks and 1 month during the fight against COVID-19 demonstrated a higher job satisfaction than those who had worked for over a month. Regression analysis suggested that the longer the anti-epidemic work duration, the lower the job satisfaction, which could be attributed to the physical and mental fatigue of medical staff after extensive hours of work (38). Therefore, during public health emergencies, the government can ensure the protection and safety of frontline medical staff as well as their physical and mental health by introducing a shift system reasonably and replacing members of the medical team frequently.

In this study, daily sleep duration was found to be a positive influencing factor for job satisfaction: the longer the sleep duration of an individual, the higher their job satisfaction. As sleep duration is a primary indicator of the quality of life, insufficient sleep will affect a person's mental status and can cause serious health problems (46). Facing heavy workload and work stress, frontline anti-epidemic medical staff particularly need adequate sleep to relieve fatigue and can be refreshed themselves in preparation for the next day (47).

Linear regression analysis showed that frontline self-participant demonstrated a substantially higher job satisfaction than those arranged by hospitals (*P* < 0.001) (48). Despite the dangerous and challenging working environment, the majority of the frontline medical staff in our study took the initiative to sign up for the prevention and control of COVID-19. They contributed to the country and society during the fight by proactively assuming the responsibilities and duties of patient treatment. Compared with the medical staff who is arranged by his or her hospital, they demonstrated higher working enthusiasm and were more likely to accept and adapt to the frontline working environment, which improved their job satisfaction.

## Limitation

There are some limitations to this study. The first one is the limited investigated sample size. However, as the research participants were medical staff of three medical teams that were sent to Hubei to aid the prevention and control of COVID-19, as well as the medical staff of a designated hospital, the results could not reflect all the job satisfaction status of frontline medical staff across China. Therefore, if the results of this study are to be verified nationally, further surveys, which contain different stages of an emergency as well as economies and related policies of different areas, should be conducted. The second limitation lies in the observation time of this study. Thirdly, this study is a cross-sectional study, which is descriptive and does not consider the time sequence of exposure (various possible factors) and outcome (job satisfaction), so it is difficult to infer the causal relationship between exposure and outcome. Furthermore, the study omitted some other potentially relevant variables such as organizational factors, including work schedule (day or night work) (49), organizational support (50, 51), the perceived oppressiveness of the organization (52), opportunity to use abilities (53), etc. If organizational factors are included in the study design, on the one hand, since job-related stress is known to have an impact on job satisfaction, some positive organizational factors such as job support can alleviate employee stress and further improve job satisfaction. The greater the opportunity to function in an organization, the higher the likelihood of job satisfaction. On the other hand, negative organizational factors such as perceived oppressiveness of the organization and night work schedule may reduce job satisfaction.

## Conclusions

The job satisfaction of the 455 participating medical staff who worked at the frontline of the prevention and control of COVID-19 was at a “relatively decent” level, higher than that reported in previous surveys among medical staff. Participants demonstrated higher job satisfaction in terms of relationships with colleagues, anti-epidemic work fulfillment, and work recognition. While participants showed lower satisfaction in terms of anti-epidemic work intensity, salaries compared with the workload and working environment, and conditions. Education, work experience, anti-epidemic work duration, daily sleep duration, and the form of participation were factors influencing their job satisfaction.

Based on the results, we recommend that related management departments and managers improve the job satisfaction of frontline medical staff by developing specific policies for medical staff in similar public health emergencies. The form of participation, prioritizing the self-fulfillment needs of medical staff with high education levels and strengthening the emergency response and practical operation training of junior staff. Meanwhile, efforts should be made to provide medical staff with psychological interventions ensure their normal sleep and rest times, meet their reasonable demands, and so on.

This study provides references for improving the job satisfaction of frontline medical staff and accelerating the construction of emergency response teams during public health crises in the future.

## Data Availability Statement

The raw data supporting the conclusions of this article will be made available by the authors, without undue reservation.

## Ethics Statement

The studies were reviewed and approved by the Ethics Committee of Xiangya School of Public Health of Central South University.

## Author Contributions

XY, YZ, HX, XZ, and JH contributed to the study design and acquisition of research data. YL conducted the data analysis. XY and YZ drafted the manuscript. CH contributed to language control and revised the manuscript. All authors contributed to the improvement of the manuscript and approved the final version for publication.

## Conflict of Interest

The authors declare that the research was conducted in the absence of any commercial or financial relationships that could be construed as a potential conflict of interest.
